# Comparing Genomic Prediction Models by Means of Cross Validation

**DOI:** 10.3389/fpls.2021.734512

**Published:** 2021-11-19

**Authors:** Matías F. Schrauf, Gustavo de los Campos, Sebastián Munilla

**Affiliations:** ^1^Facultad de Agronomía, Universidad de Buenos Aires, Buenos Aires, Argentina; ^2^Animal Breeding & Genomics, Wageningen Livestock Research, Wageningen University & Research, Wageningen, Netherlands; ^3^Departments of Epidemiology, Biostatistics, Statistics, and Probabilty, Institute for Quantitative Health Science and Engineering, Michigan State University, East Lansing, MI, United States; ^4^Instituto de Investigaciones en Producción Animal (INPA), CONICET-Universidad de Buenos Aires, Buenos Aires, Argentina

**Keywords:** genomic selection, cross validation, plant breeding, genomic models, model selection

## Abstract

In the two decades of continuous development of genomic selection, a great variety of models have been proposed to make predictions from the information available in dense marker panels. Besides deciding which particular model to use, practitioners also need to make many minor choices for those parameters in the model which are not typically estimated by the data (so called “hyper-parameters”). When the focus is placed on predictions, most of these decisions are made in a direction sought to optimize predictive accuracy. Here we discuss and illustrate using publicly available crop datasets the use of cross validation to make many such decisions. In particular, we emphasize the importance of paired comparisons to achieve high power in the comparison between candidate models, as well as the need to define notions of relevance in the difference between their performances. Regarding the latter, we borrow the idea of equivalence margins from clinical research and introduce new statistical tests. We conclude that most hyper-parameters can be learnt from the data by either minimizing REML or by using weakly-informative priors, with good predictive results. In particular, the default options in a popular software are generally competitive with the optimal values. With regard to the performance assessments themselves, we conclude that the paired k-fold cross validation is a generally applicable and statistically powerful methodology to assess differences in model accuracies. Coupled with the definition of equivalence margins based on expected genetic gain, it becomes a useful tool for breeders.

## 1. Introduction

In essence, genomic models relate genotypic variation as present in dense marker panels to phenotypic variation in a given population. These models were first introduced in breeding (Meuwissen et al., 2001) as a change of paradigm with respect to traditional marker assisted selection. They are currently used to accelerate genetic gain in many plant breeding programs with the focus placed on improving predictive ability while remaining agnostic to the causative nature of the genotype-phenotype relation. When fitting genomic models to data, practitioners need to make multiple decisions, sometimes without a clear guide or approach on how to take them. Besides the decision of choosing which model to use among the increasing number available (Whittaker et al., 2000; Meuwissen et al., 2001; VanRaden, 2008; de Los Campos et al., 2010; Habier et al., 2011; Ober et al., 2015), the practitioners also need to make many minor choices for those parameters which are not directly estimated by the data (so called “hyper-parameters”). When the focus is placed on predictions, as it is usual with genomic models, most of these decisions are made in a direction sought to optimize predictive accuracy. This accuracy is usually estimated in practice by means of cross validations.

Because of the impact of the prediction accuracy on genetic gain, many benchmarks have been done seeking to compare such accuracies among competing models. Most conclude that there is no better model in general (Heslot et al., 2012), with the recommendation that practitioners evaluate the entertained models with their own data and for the specific prediction tasks at hand (Azodi et al., 2019). The present work illustrates how the different performance assessments and comparisons can be made with cross validations, with a focus placed on both identifying differences of practical relevance and the decision making required for model selection and hyper-parameter tuning. We emphasize the importance of conducting paired cross validations to achieve higher statistical power, and propose the use of equivalence margins to identify the differences in accuracy which are relevant in practice.

With these goals in mind, the present work is organized as follows: we first assess the predictive ability of G-BLUP (VanRaden, 2008), probably the most known genomic model, in a well studied dataset, where we discuss the general aspects of cross validation. We then move on to the comparison of predictive abilities, which we first use to select the model complexity of BayesA by tuning the prior average variance of marker effects. We then consider general hyper-parameter tuning and evaluate the impact each hyper-parameter has on the accuracy for a variety of models. We explore general model comparisons, and describe tools to identify relevant differences in accuracy. To show an assessment of accuracy differences across multiple datasets we explore whether a pattern observed in the previous section can be generally extrapolated. We close with some final remarks.

## 2. Materials and Methods

### 2.1. The Datasets

In the present work we used public datasets from three main crops: wheat, rice and maize. The first dataset consists of 599 CIMMYT wheat lines, genotyped with 1,279 DArT markers. The wheat lines were grown in four different environments and grain yield was recorded for each line and each environment (Crossa et al., 2010). This dataset is easily accessed from the R package BGLR (Perez and de los Campos, 2014) and its relatively small size allowed us to assess a greater number of models and parameter combinations.

The remaining two datasets include both more lines and genotyping by sequencing. They were included in the last section ‘Comparison across datasets’. The rice dataset consists of 1,946 lines, which were genotyped by the 3,000 Rice Genomes Project (Wang et al., 2018). We used four quantitative traits available on a high number of lines: grain weight, width and length and the date on which 80% of the plants are heading. Finally, the maize dataset consists of lines from the “282” Association Panel and the NAM population. These lines were genotyped by the project “Biology of Rare Alleles in Maize and its Wild Relatives” (Glaubitz et al., 2014). For these lines we used four contrasting traits: the germination count, the number of leaves, the days to tassel, and plant height.

### 2.2. The Genomic Models

In the current work we assessed the performance of a variety of statistical models coming from two families of common use in genomic selection. The first family of models we considered is the so-called “Bayesian alphabet” (Gianola et al., 2009) and consists of regressions of phenotypes on markers. The second family comprises models that use the markers to build genomic relationship matrices (GRM), used in turn to model the covariance among genetic effects. These latter models stem from the linear mixed models tradition in breeding, which can be traced back to Henderson (cf. Henderson, 1984).

Models of the first family, the Bayesian alphabet, are usually formulated in a hierarchical structure of the form:


$$\begin{array}{l} y=\mu +X\beta +\varepsilon \\ \beta \sim F\left(\Theta \right) \end{array}$$

where *y* is an n-length vector of trait phenotypes, μ is the vector of means (possibly dependent on fixed-effect predictors), *X* is an incidence matrix of the marker effects in the p-length vector β, and ε is an n-length vector of normally distributed errors (with environmental and unmodelled effects confounded). As the number of markers (p) typically exceeds the number of different genotypes (n), the regression equation is over-parameterized. Bayesian alphabet models deal with this “*n* ≪ *p*” situation by assuming a prior distribution F(Θ) for the marker effects. Each model is distinguished by the distribution of such priors, which we briefly describe in Box 1.

Box 1 **Priors of marker effects in models of the “Bayesian alphabet” used in this work.**

**rrBLUP:** β_*j*_ ~ normal distribution

$\beta_{j}\sim N\left(0,\sigma_{\beta }^{2}\right)$

see Whittaker et al. (2000),

**BayesA:** β_*j*_ ~ scaled t-student distribution

$\beta_{j}|\sigma_{\beta_{j}}^{2}\sim N\left(0,\sigma_{\beta_{j}}^{2}\right)$

$\sigma_{\beta_{j}}^{2}\sim$ Scaled-inv-χ^2^(ν, *S*)

see Meuwissen et al. (2001),

**BayesB:** β_*j*_ ~ spike-slab with scaled t-student distribution

$\beta_{j}|\sigma_{\beta_{j}}^{2}\sim N\left(0,\sigma_{\beta_{j}}^{2}\right)$

$\sigma_{\beta_{j}}^{2}=0$, with probability π

$\sigma_{\beta_{j}}^{2}\sim$Scaled-inv-χ^2^(ν, *S*), with probability (1 − π)

see Meuwissen et al. (2001),

**BayesC:** β_*j*_ ~ spike-slab with normal distribution

β_*j*_ = 0, with probability π

$\beta_{j}\sim N\left(0,\sigma_{\beta }^{2}\right)$, with probability (1 − π)

see Habier et al. (2011).

Note that, after Gianola et al. (2009), it is usual to marginalize the marker effect distribution over all other marker-specific parameters in the prior. As an example, by marginalizing over the marker-specific variance ($\sigma_{\beta_{j}}^{2}$), BayesA is usually characterized as having a scaled t-student distribution for the markers effects priors. Also in the literature, priors with a mass probability at zero are called spike-slab (like those used in BayesB and BayesC). In this work we do not interpret these Bayesian priors as statements of belief, but rather as regularization devices (Gelman and Shalizi, 2013). They stabilize estimates and predictions by making fitted models less sensitive to certain details of the data, and thus alleviate the over-parameterization problem in genomic models.

The second family of models considered consists of mixed linear models, where marker information is used to build-up relationship matrices. All these models may be specified as follows:


$$\begin{array}{l} y=X\beta +\underset{i}{\sum }Z^{\left(i\right)}u^{\left(i\right)}+\varepsilon \\ u^{\left(i\right)}\sim N\left(0,G^{\left(i\right)}\sigma_{u^{\left(i\right)}}^{2}\right)\\ \varepsilon \sim N\left(0,I\sigma_{e}^{2}\right) \end{array}$$

where *y* is an n-length vector of trait phenotypes, *X* is an incidence matrix of the fixed effects in β, each *Z*^(*i*)^ is an incidence matrix of the individual genetic values in the n-length vector *u*^(*i*)^, and ε is an n-length vector of errors (with environmental and unmodelled effects confounded). Each model is distinguished by different (often one, possibly many) genomic relationship matrices [*G*^(*i*)^] described in Box 2. These genomic relationship matrices (GRMs) specify the covariance structure of the genetic values.

Box 2 **Genomic relationship matrices and the mixed models which use them.**

**G-BLUP:**

*G* ∝ (*M* − 2 · 1*P*)(*M* − 2 · 1*P*)′

see VanRaden (2008),

**EG-BLUP:**

*H* ∝ *G* ⊙ *G*, (where ⊙ is the hadamard product)

see Ober et al. (2015); Martini et al. (2016),

**Categorical Epistasis:**

$Cm_{ij}\propto \frac{1}{p}\cdot \underset{k}{\sum }\left[M_{ik}=M_{jk}\right]$, (where [*proposition*]: = 1 if true, else 0)

$Ce\propto \frac{1}{2}\left(Cm⊙Cm+Cm\right)$

see Martini et al. (2017),

**Gaussian Kernel:**

$D_{ij}=\frac{1}{p}\cdot \underset{k}{\sum }|M_{ik}-M+jk{|}^{2}$ (alternatively, *D*_*ij*_ = *G*_*ii*_ + *G*_*jj*_ − 2*G*_*ij*_)

*K*_*ij*_ ∝ *exp*(*D*_*ij*_/*h*), (elementwise exponentiation)

see de Los Campos et al. (2010) and Alves et al. (2019),

Symbols:

*M*_*ik*_: allele incidence matrix

*P*: allele frequencies

GRMs are defined up to a multiplicative constant, which can be absorbed into the corresponding variance parameter ($\sigma_{g}^{2}$) in the mixed model.

### 2.3. Cross Validations for Model Assessment

In this work we used k-fold cross validation in order to assess each model's predictive performance (cf. Friedman et al., 2001). This procedure consists of dividing a dataset with n cases (including both phenotypes and genotypic information) into a number of folds (k) of approximately equal size. Data in k-1 folds are used for training the model to predict phenotypes in the remaining fold (the testing fold), given the realized genotypes. The prediction task is repeated using one fold at a time for testing, and overall results are then combined. When the partitioning into folds is repeated, say r times, the procedure is called an r-replicated k-fold cross validation.

An important aspect in the design of a cross validation test is to define an appropriate error measure to be minimized. In this regard, a reasonable choice would be the mean square error (MSE), which penalizes every departure in predictions from the observed values. However, in the context of breeding this measure can be too strict, as any constant or scaling factor afflicting all predictions will inflate the MSE but will not change the ranking. Instead, breeders have focused on estimating the predictive accuracy (accuracy, for short), measured as the correlation between predictions and observations.

In practice, genetic values are usually the ultimate prediction targets rather than phenotypes. To account for this, the accuracy can be re-scaled dividing by the square root of a heritability estimate (notice it is important to use the same heritability estimate for all accuracies compared to each other). It is possible, though, to go one step further and directly focus on estimating the expected genetic gain, which is easily obtained if we assume truncation selection. We used this new re-scaling into expected genetic gain in the section “Comparison across datasets” (in results and discussion). The scaling factor can be easily derived from the standard genetic gain formula (cf. Falconer and Mackay, 1996, in “Response to selection”):


$$\begin{array}{l} \Delta G=i_{q}\cdot r_{g}\cdot \sigma_{G}\\ \Delta G=i_{q}\cdot \left(r_{ph}/h\right)\cdot \sigma_{G}\\ \Delta G=i_{q}\cdot r_{ph}\cdot \left(\sigma_{P}/\sigma_{G}\right)\cdot \sigma_{G}\\ \Delta G=i_{q}\cdot r_{ph}\cdot \sigma_{P}\\ \Delta G/\sigma_{P}=i_{q}\cdot r_{ph} \end{array}$$

where *r*_*g*_ is the predictive accuracy with respect to the (unobserved) true genetic values, *r*_*ph*_ is the predictive accuracy with respect to phenotypes, *i*_*q*_ is the selection intensity (i.e., the mean of a standardized Normal distribution truncated at the *q* selection quantile), and Δ*G*/σ_*P*_ is the estimate of genetic gain (in phenotypic standard deviations). This genetic gain measure is quite simplistic (as it assumes selection by truncation and random mating), but on the other hand is easily interpretable and of practical relevance.

There are two further important issues with regard to cross validations. One concerns the partitioning between training and testing sets. While here we always used random partitions, in specific cases it can be more appropriate to use other schemes such as splitting the dataset in generations, half-sib families or sub-populations. Also, in the context of multi-trait models, available information about the different traits can vary between selection candidates at the time of prediction; thus blurring the distinction between training and testing sets (Runcie and Cheng, 2019).

The second issue concerns whether we are interested in estimating the accuracy conditional on the available training set or as a marginal expectation; i.e., averaged over different possible training sets. In the context of a breeding plan, where the genomic model gets updated with new data, the marginal predictive accuracy might be the more appropriate. Fortunately, this is the version of the accuracy which is thought to be better estimated by a k-fold cross validation, while a leave-one-out cross validation might be better tailored to estimate the conditional predictive accuracy (cf. Friedman et al., 2001, section 7.12). In summary, the cross validation should be designed to accurately simulate the real-world usage of the genomic model.

### 2.4. Paired Cross Validations for Model Comparison

The problem of identifying a superior model is different from the performance assessment task such as discussed in the previous section. While one could conduct model selection simply by choosing the model with the highest estimated performance, it is important to take the variability of those estimates into account, as well as to provide some control for error probabilities according to statistical established practice. When applying an r-replicated k-fold cross validation procedure, variability in the performance estimates arises from the r replicates and the k folds. However, using the variability estimate of each assessment independently (surprisingly an extended practice) ignores that most variability is shared among models.

**Figure 1:**
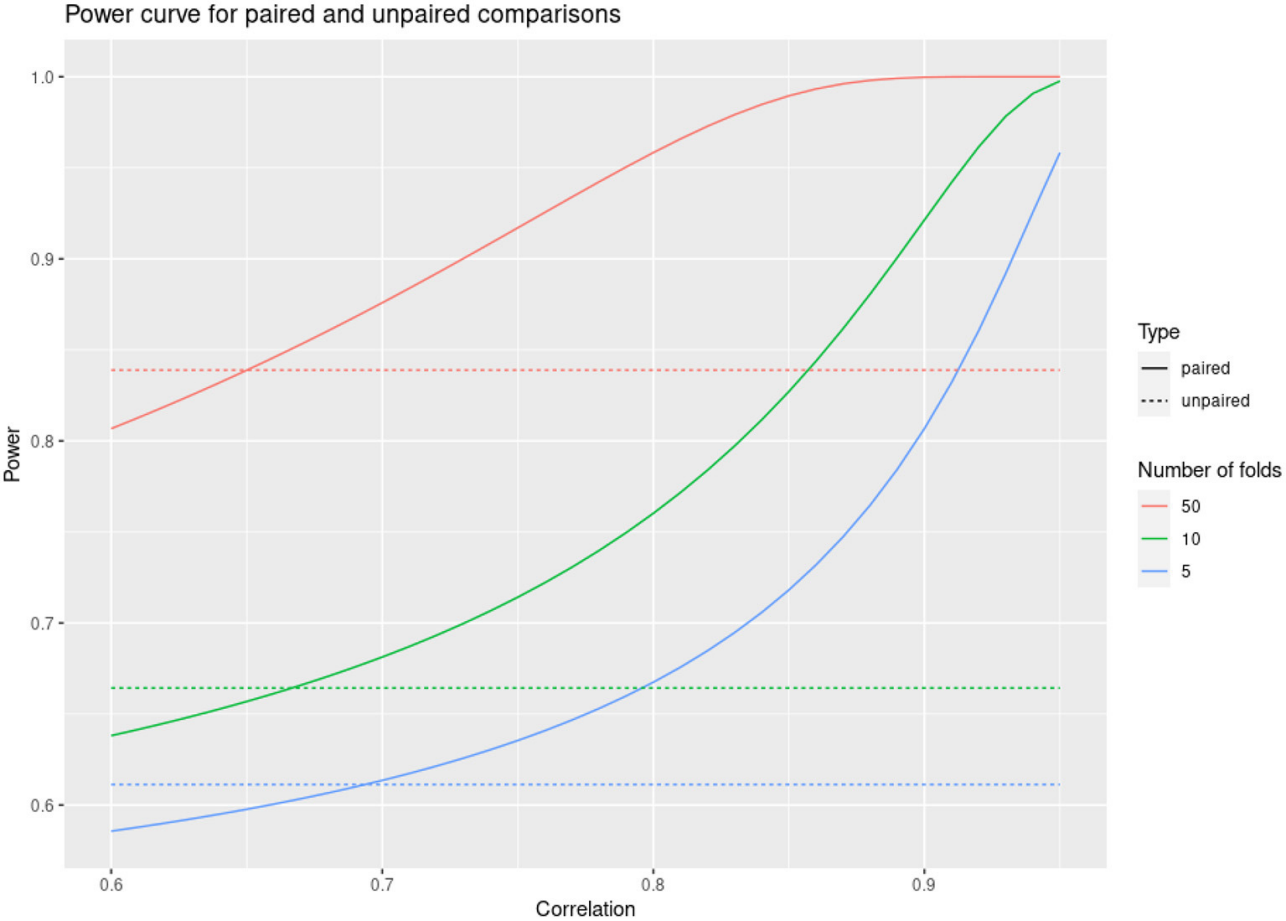
Power curve for paired and unpaired cross validations as a function of fold correlation between compared models.

A much more reasonable approach when comparing predictive accuracies between models is to perform paired comparisons within the same partitioning of folds (Hothorn et al., 2005). That is, for each fold one summarizes the difference in accuracies between the compared models rather than the individual accuracies. This often results in a huge reduction in the variance of the performance estimates, because most of the variability is usually shared across the different models. For example, if the correlation across folds of the accuracy scores for two models is over 0.8, then the variance of the estimate of the accuracy difference can be reduced five times by taking this approach, with a corresponding increase in statistical power (see Figure 1). We employed this approach in all our model comparisons.

### 2.5. Equivalence, Non-inferiority and Superiority Tests

The comparison of model accuracies using paired differences of cross validation can have high statistical power. This allows detecting with high confidence very small differences in performance. Such statistically significant differences of small magnitude can be uninteresting because they are superseded by considerations other than accuracy, or they might not be robust to any changes in the application of the models. As the saying goes, “*With great power there must also come great responsibility”*. Here it is the responsibility of practitioners to evaluate the differences, not only by the statistical ability to detect them, but also by their assessed practical relevance.

To help with this task, we propose defining an “equivalence margin” [-Δ, Δ] within which model performances are deemed equivalent in practice. These kinds of equivalence margins are standard used in clinical studies (e.g., Da Silva et al., 2009) but, to the best of our knowledge, their use is not widespread in plant breeding or the agricultural and environmental sciences in general. Then, in addition to the conventional test for statistical differences (sd)

- *H*_0_:*d* = 0,

we use the machinery of statistical tests to provide assertions on the practical relevance of these differences with some degree of error control. Specifically, by conducting tests of

- Equivalence (eq), *H*_0_:|*d*| > Δ
- Non-inferiority (noi), *H*_0_:*d* < −Δ
- Superiority (sup), *H*_0_:*d* < Δ

The hypothesis for these tests are illustrated in Box 3.

Box 3 **Representation of the null (*H*_0_) and alternative (*H*_1_) hypothesis for specific tests:**

sd:    < -------)[](--------->

eq:    < ----](-----)[------->

noi:   < ----](-------------->

sup:   < ------------](------>

       < ----|---+---|------->

          - Δ   0  +Δ

Null hypotheses represented in gray, alternative hypotheses in black.

We can use these tests to assess the practical relevance of differences in predictive accuracy. With the result of these tests we can produce labels similar to the “significance letters”, which we argue have some advantages with regard to their interpretation:

- Equivalence letters: models sharing the same letter have an accuracy difference confidently within the equivalence margin (and thus are deemed equivalent for practical purposes).
- Non-inferiority ranking: models with the same or higher ordinal are confidently non-inferior (the accuracy difference is within or above the equivalence margin).
- Superiority ranking: models with higher ordinal are confidently superior (the accuracy difference is above the equivalence margin).

To build these labels we use directed graphs where the nodes are the models compared and they are connected by an edge if the null hypothesis for the comparison is rejected.

- Equivalence letters: One letter is assigned to each clique of the graph (which is effectively an undirected graph due to the reflexivity of the equivalence test).
- Non-inferiority and Superiority rankings: The rankings are built from the consensus ordering of all possible topological orders for their respective directed graphs.

These algorithms are similar in nature to those used by statistical software to compute the traditional significance letters. We note, though, that traditional significance letters should not be interpreted as meaning that elements with the same letter are equivalent which, instead, is the correct interpretation for the equivalence letters built with the construction above. Finally, we would like to mention that the hypothesis tests covered in this section have a general scope of application and are not restricted to the comparisons of model performance.

### 2.6. Software

The GRMs were built with custom code in the Julia programming language (Bezanson et al., 2017), available upon request from the corresponding author. The remaining analyses were done in the R programming language (R Core Team, 2021). In particular, the “Bayesian alphabet” models were fitted with the BGLR package (Perez and de los Campos, 2014) and the mixed models were fitted with the EMMREML package (Akdemir and Godfrey, 2015). We used the bootstrap utilities from the package “boot” (Davison and Hinkley, 1997; Canty and Ripley, 2021). Finally, the functions for the analysis of cross validation results and equivalence margin testing were organized into the R package “AccuracyComparer” (available at https://github.com/schrauf/AccuracyComparer).

## 3. Results and Discussion

### 3.1. Model Predictive Ability Assessment

**Figure 2:**
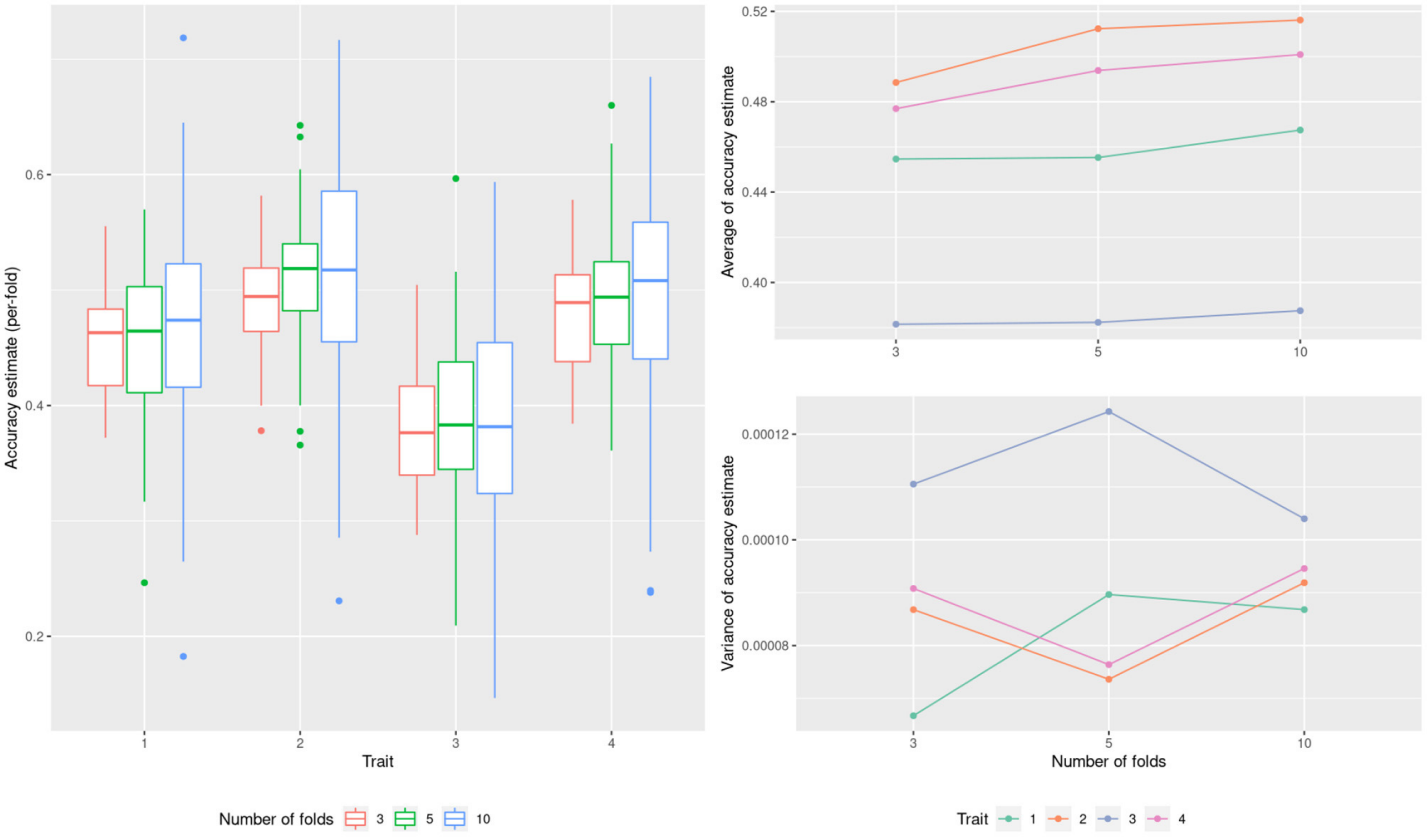
Model performance estimation for the wheat dataset with varying number of cross-validation folds.

As a starting point and to illustrate the use of the cross validation technique we estimate the ability of a G-BLUP model to predict CIMMYT wheat yield across four environments. Figure 2 shows the accuracies estimated by the K-fold cross validation when using different numbers of folds (K = 3, 5 and 10). The means bias downward for a smaller number of folds (panel b) but the effect is small. For the variance of the estimate there is no clear tendency (panel c). This is because of two competing effects that balanced out. For one, as the size of the testing set increases (less folds), this reduces the variance of the estimate at each fold (panel a). In the opposite direction, as the number of folds increases, the variance of the whole cross validation estimate reduces. To estimate the marginal predictive error, both 5-fold and 10-fold seem reasonable choices, with smaller bias than 3-fold cross validation and similar variances. As briefly mentioned in materials and methods, a greater number of folds should not be used unless the goal is to estimate the conditional predictive accuracy. In all the following sections we used 10-fold cross validations.

### 3.2. Model Selection

#### 3.2.1. Model Complexity and Penalization Parameter

Most genomic models have some penalization parameters which regulate how flexibly the model adjusts to sample observations. Finding an optimal value for these parameters is a typical task for cross validation. Alternatively, these penalization parameters can be learnt from the data by either minimizing the REML criterion in mixed models (where the penalization parameters are variance components, see Bates et al., 2014) or by using non or weakly-informative priors in Bayesian alphabet models.

As an example, take the case of BayesA, where model penalization is mainly controlled by the scale parameter of the chi-squared distribution (S, in Box 1), which in turn determines the a priori average variance of the marker effects ($𝔼\left[\sigma_{\beta_{j}}^{2}\right]$). With BGLR we can choose the value of this parameter by specifying the proportion of phenotypic variance a-priori expected to be explained by the marker effects (in the following “$R_{geno}^{2}$,” see Perez and de los Campos, 2014), which allows for an easier interpretation.

**Figure 3:**
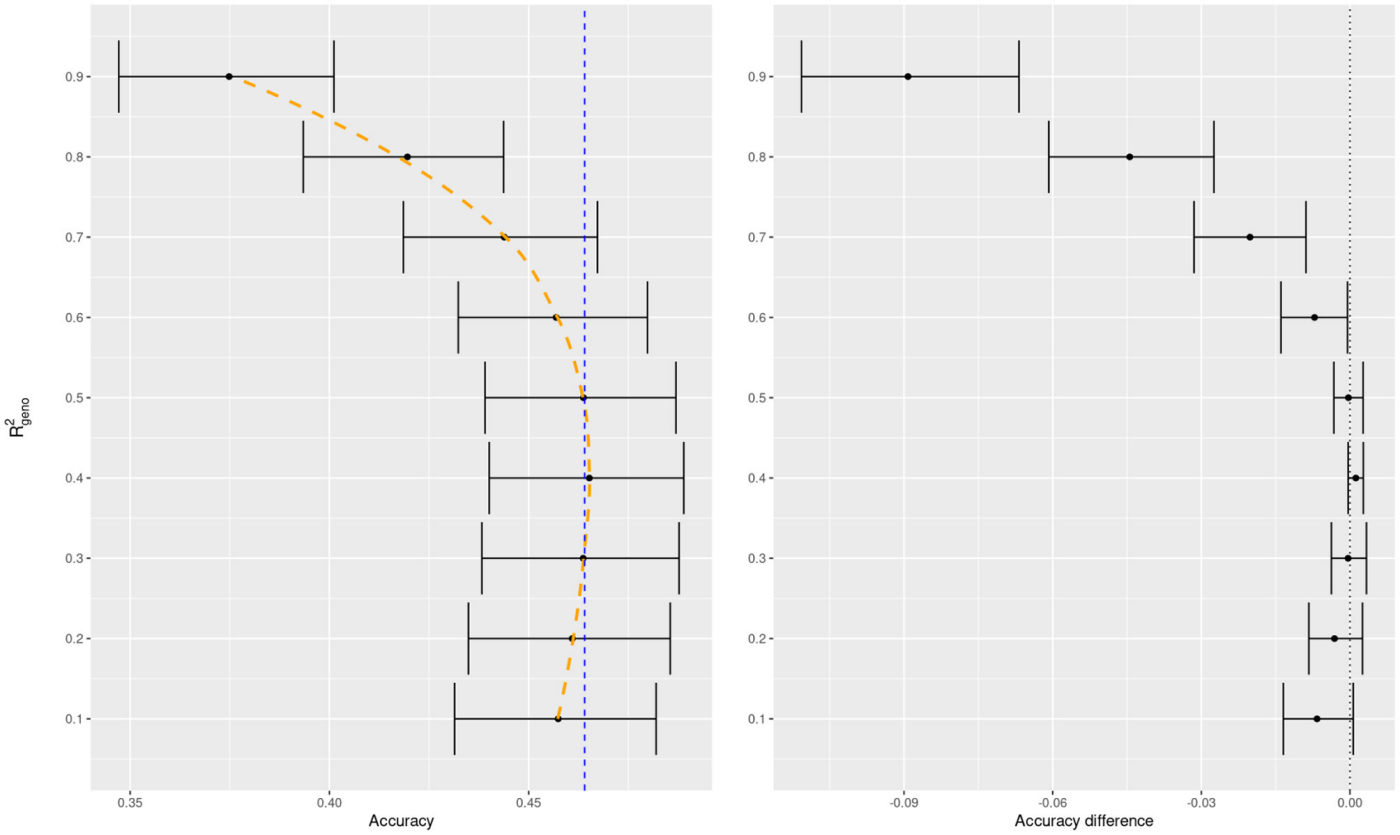
BayesA predictive accuracy as function of prior mean of $R_{geno}^{2}$ for trait 1 of the wheat dataset. Average predictive accuracy of the default model in BGLR (a weakly-informative prior for $R_{geno}^{2}$) in dashed blue for the left panel. All accuracy differences in the right panel are taken with respect to the default model.

To illustrate the usefulness of cross validation to elicit these parameters, we conducted a 10-fold cross validation for BayesA with a grid of values for $R_{geno}^{2}$ when fitting wheat yield data. From this we can observe a textbook accuracy curve which results from the classical bias-variance tradeoff (cf. Friedman et al., 2001). Starting with low values of $R_{geno}^{2}$ we have rigid models, whose accuracies improve with increasing $R_{geno}^{2}$, until the models begin to overfit and the accuracy rapidly deteriorates (Figure 3, left panel). This resulted in an intermediate optimal value.

In addition, we compared the difference in accuracies of the specific variance proportions in a model with a weakly-informative prior which is the default in BGLR (Figure 2, right panel). We can see that the model which learns the variance proportion from the data performs competitively with the best pre-specified values of $R_{geno}^{2}$. We know that REML is a sound criterion for learning variance components (Thompson, 2019) and known theoretical results match REML estimates to the mode of the posterior distribution of the parameter when a non-informative prior is set in a Bayesian model (cf. Sorensen and Gianola, 2002, chapter 9). It is possible then, that the soundness of REML applies not only to Bayesian mixed models but also, at least approximately, to other Bayesian regressions when using weakly informative priors.

#### 3.2.2. Hyper-Parameter Tuning

**Figure 4:**
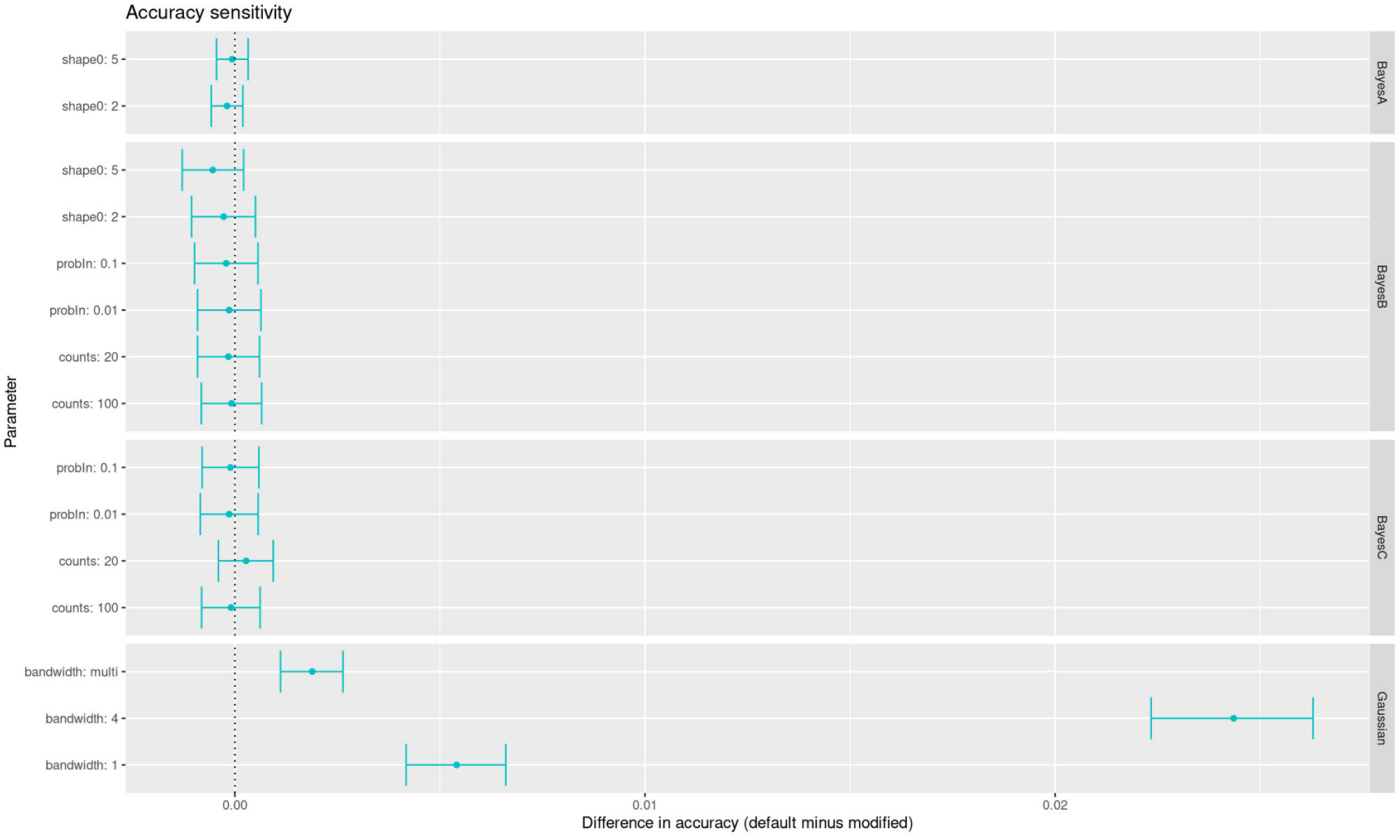
Accuracy sensitivity of multiple models to changes in the values of their hyper-parameters (alternative values compared with respect to defaults in BGLR software).

Beside penalization parameters, there are many hyper-parameters without a clear impact on accuracy in the priors of Bayesian regressions. On the other hand, mixed linear models have fewer ones, a notable exception being the bandwidth of the Gaussian kernel. Here we have summarized the impact of many of those hyper-parameters affecting the models when fitting the wheat yield data. For each parameter we show the change in accuracy with respect to the default value in BGLR (Figure 4). We can see that, of these parameters, only changes in the kernel's bandwidth impact the accuracy with a statistically significant change. An alternative to arbitrary choices is to use multiple kernels in the same model, each kernel with a different bandwidth (Alves et al., 2019). These multi-kernel models have the ability to weight the contribution of each kernel, with results close to optimal.

#### 3.2.3. General Model Comparison

**Figure 5:**
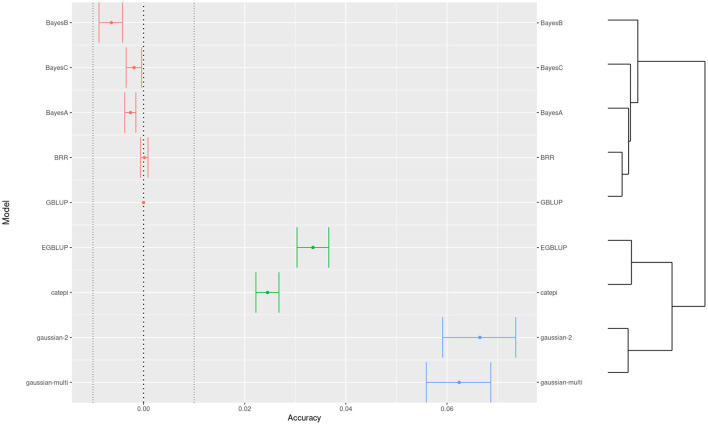
Comparison of predictive accuracies across models for the trait 1 of the wheat dataset. Colors show a 3-group clustering of the models based on the accuracy with a hierarchical clustering on the right side.

Beyond setting hyper-parameters, which in our exploration resulted in minor changes in accuracy, the practitioners may want to compare between distinctly different models. Here we see how one may proceed to compare between more than two models, when they are not necessarily organized by a specific parameter. We used clustering to help interpretation, and we chose the G-BLUP as a reference model to compare accuracy differences (Figure 5). Still referring to wheat yield, BayesB performed the worst and the Gaussian kernel methods the best. Because of high power, we defined an “equivalence margin” to identify the relevant differences. This allowed us to identify easily interpreted groups of statistically equivalent models (Table 1). Concretely, the 3 equivalent groups were the additive models (A), models with only pairwise interactions between markers (B), and finally the models with higher order interactions (C). We explore further this relation between marker interactions and predictive performance in the following section.

**Table 1:** General model comparison for the wheat dataset.

	**Accuracy difference**			**Hypothesis tests**		
**Model**	**Mean**	**Lower**	**Upper**	**sd**	**eq**	**Sup**
BayesB	–0.006	–0.009	–0.004	a	A	1
BayesC	–0.002	-0.003	0.000	b	A	1
BayesA	–0.003	-0.004	–0.002	b	A	1
GBLUP	0.000	-	-	c	A	1
BRR	0.000	–0.001	0.001	c	A	1
Catepi	0.025	0.022	0.027	d	B	2
EGBLUP	0.033	0.030	0.037	e	B	2
Gaussian-multi	0.062	0.056	0.069	f	C	3
Gaussian-2	0.067	0.059	0.074	f	C	3

*Accuracy differences measured with respect to the G-BLUP model. Hypothesis tests (sd, statistical differences; eq, equivalence; sup, superiority). Meaning of letters and rankings in section 2.5*.

#### 3.2.4. Comparison Across Datasets

**Figure 6:**
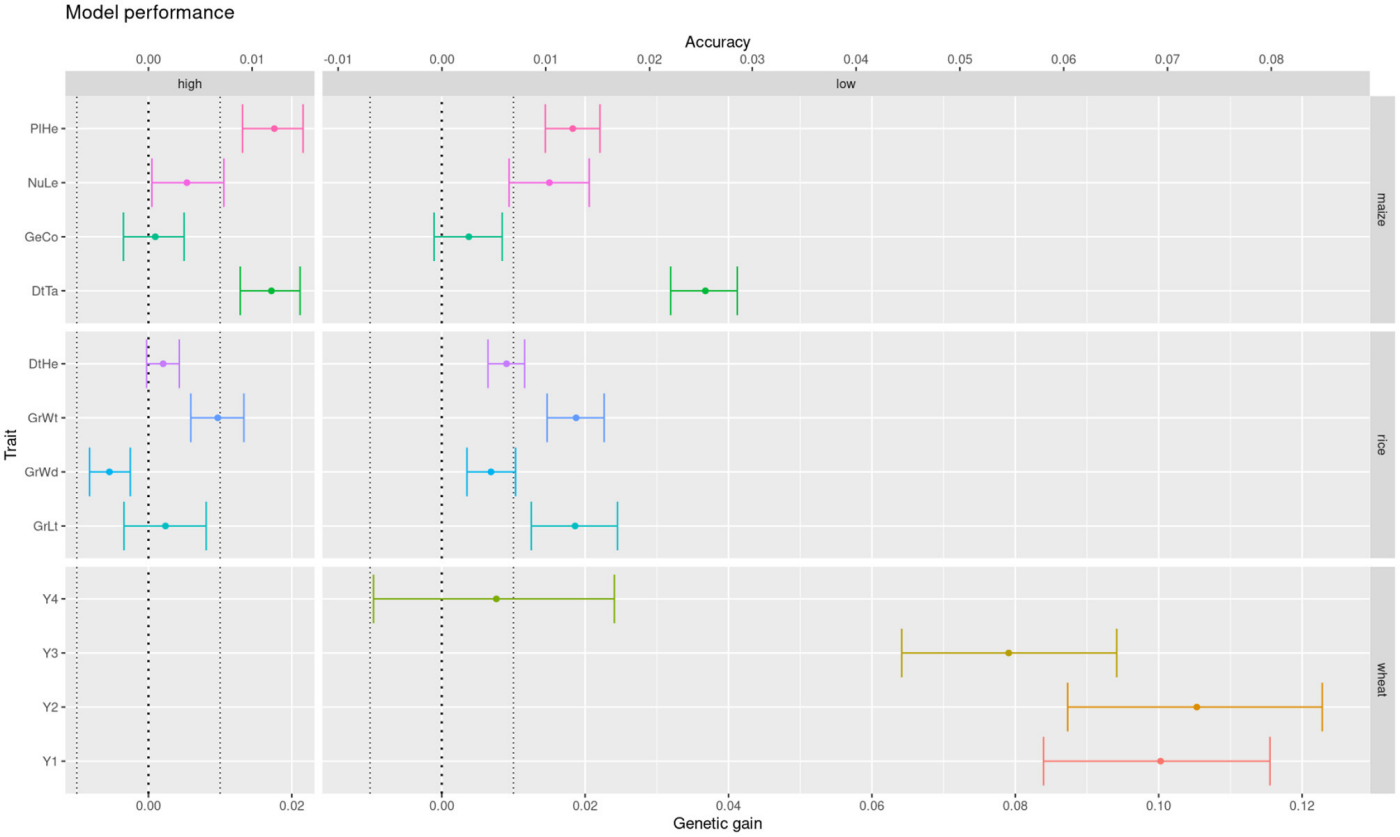
Difference in expected genetic gain from using a Gaussian kernel with respect to a GBLUP prediction model, for low and high panel densities, across multiple crops and traits (color coded for legibility).

Sometimes we need to compare models across different datasets or prediction tasks. For instance, we would like to see here if the difference between additive and epistatic models observed in the previous section is particular to the wheat dataset. Schrauf et al. (2020) showed that marker density could be a relevant factor for the advantage of models with marker interactions. Recall that wheat lines were genotyped at low density with DaRTs, whereas rice and maize lines were sequenced. So we compared the models performance across datasets with both low and high density marker panels from these latter species. Also, to assess the relevance of the differences in accuracy, we converted them to differences in expected genetic gain (assuming truncating selection of the highest 10% genetic values). This scale could help practitioners in deciding on relevant equivalence margins for the equivalence, non-inferiority and superiority hypothesis tests. We can see that the advantage for the Gaussian kernel over the GBLUP model observed for wheat in the previous section is much less clear for the maize and rice datasets (Figure 6). Further, the improvements that can be observed are reduced when going from a low density marker panel to a high density one. In particular, the traits where the models were statistically equivalent rose from under 10% with low density panels to half at high density panels (Table 2). These results are in accordance with the phenomena of phantom epistasis (Schrauf et al., 2020).

**Table 2:** Difference in expected genetic gain from using a Gaussian kernel with respect to a GBLUP prediction model, for low and high panel densities.

			**Genetic gain difference**			**Hypothesis tests**			
**Markers**	**Species**	**Trait**	**Mean**	**Lower**	**Upper**	**sd**	**eq**	**noi**	**Sup**
High	Maize	Days to tassel	0.017	0.013	0.021	*		*	*
		Germination count	0.001	–0.003	0.005		*	*	
		Number of leaves	0.005	0.000	0.011	*		*	
		Plant height	0.018	0.013	0.022	*		*	*
	Rice	Grain length	0.002	–0.003	0.008		*	*	
		Grain width	–0.005	–0.008	–0.003	*	*	*	
		Grain weight	0.010	0.006	0.013	*		*	
		Days to heading	0.002	0.000	0.004		*	*	
						0.63	0.5	1	0.25
Low	Maize	Days to tassel	0.037	0.032	0.041	*		*	*
		Germination count	0.004	–0.001	0.008		*	*	
		Number of Leaves	0.015	0.009	0.021	*		*	
		Plant height	0.018	0.014	0.022	*		*	*
	Rice	Grain length	0.019	0.012	0.025	*		*	*
		Grain width	0.007	0.004	0.010	*		*	
		Grain weight	0.019	0.015	0.023	*		*	*
		Days to heading	0.009	0.006	0.012	*		*	
	Wheat	Yield 1	0.100	0.084	0.116	*		*	*
		Yield 2	0.105	0.087	0.123	*		*	*
		Yield 3	0.079	0.064	0.094	*		*	*
		Yield 4	0.008	-0.010	0.024			*	
						0.83	0.08	1	0.58

*Hypothesis tests (sd, statistical differences; eq, equivalence; noi, non-inferiority; sup, superiority), and proportion of models with rejected nulls for high and low panel densities. Asterisks indicate rejected nulls for the corresponding test (see Box 3)*.

### 3.3. Final Remarks

In the present work we explored a variety of aspects related to the performance assessment of genomic models via cross validations. We identified several strategies which can help practitioners avoid arbitrary decisions when implementing a particular genomic prediction model. For instance, many hyper-parameters can be effectively learnt from the data by either minimizing REML or by using weakly-informative priors. In particular, the default values of those hyper-parameters in the software used (BGLR) are generally competitive with the optimal values. An exception is the choice of bandwidth in a gaussian kernel, for which different values can result in qualitatively different predictive performances of the model. For this particular case we recommended the use of multi-kernel models.

Throughout the work we used paired cross validations to compare methods. This was motivated by the fact that cross validation estimates are greatly correlated between models. While the cross validation estimate of the performance of a model can have a high variability, the estimate of the difference in performance between two models is usually much more precise and allows for their comparison with higher statistical power. We concluded that paired k-fold cross validations result in a generally applicable and statistically powerful methodology to assess differences in model accuracies.

Finally, we introduced the idea of equivalence margins as a means to identify when those significant differences have practical relevance for decision making and model selection. This is important because with high statistical power small differences become detectable, which might not be of interest, or might not be robust to even small changes between the validation and the application of the models. We suggest to couple the tool of equivalence margins, and the associated hypothesis tests, with informative performance scales for the tasks at hand. In a breeding context, such scale could be the potential genetic gain from truncation selection.

## Data Availability Statement

Publicly available datasets were analyzed in this study. This data can be found here: https://www.panzea.org/; https://doi.org/10.7910/DVN/HGRSJG; https://cran.r-project.org/web/packages/BGLR/index.html.

## Author Contributions

MS carried out the analyses and wrote the manuscript with support from SM. GC provided key ideas and revised intermediate stages. All authors discussed the results and contributed to the final manuscript.

## Funding

This publication resulted (in part) from research supported by multiple institutions: MS received funding from the National Scientific and Technical Research Council (CONICET) in the form of a doctoral scholarship. GC received funding from the National Institute for Food and Agriculture (NIFA) of the USDA (award #2021-67015-33413). SM received funding from UBACyT 2020 (proyect: 20020190200324BA).

## Author Disclaimer

The contents are solely the responsibility of the authors and do not necessarily represent the official views of the funding institutions.

## Conflict of Interest

The authors declare that the research was conducted in the absence of any commercial or financial relationships that could be construed as a potential conflict of interest.

## Publisher's Note

All claims expressed in this article are solely those of the authors and do not necessarily represent those of their affiliated organizations, or those of the publisher, the editors and the reviewers. Any product that may be evaluated in this article, or claim that may be made by its manufacturer, is not guaranteed or endorsed by the publisher.

## References

[B1] Akdemir D. Godfrey O. U. (2015). EMMREML: fitting mixed models with known covariance structures. R package version 3.1.

[B2] Alves F. C. Granato Í. S. C. Galli G. Lyra D. H. Fritsche-Neto R. de Los Campos G. (2019). Bayesian analysis and prediction of hybrid performance. Plant Methods 15, 1–18. 10.1186/s13007-019-0388-x 30774704 PMC6366084

[B3] Azodi C. B. Bolger E. McCarren A. Roantree M. de Los Campos G. Shiu S.-H. (2019). Benchmarking parametric and machine learning models for genomic prediction of complex traits. G3 9, 3691–3702. 10.1534/g3.119.400498 31533955 PMC6829122

[B4] Bates D. Mächler M. Bolker B. Walker S. (2014). Fitting linear mixed-effects models using lme4. arXiv preprint arXiv:1406.5823. 10.18637/jss.v067.i01

[B5] Bezanson J. Edelman A. Karpinski S. Shah V. B. (2017). Julia: A fresh approach to numerical computing. SIAM Rev. 59, 65–98. 10.1137/141000671

[B6] Canty A. Ripley B. D. (2021). boot: Bootstrap R (S-Plus) Functions. R package version 1.3–28.

[B7] Crossa J. Campos G. d. l Pérez P. Gianola D. Burgueno J Luis Araus J . (2010). Prediction of genetic values of quantitative traits in plant breeding using pedigree and molecular markers. Genetics 186, 713–724. 10.1534/genetics.110.118521 20813882 PMC2954475

[B8] Da Silva G. T. Logan B. R. Klein J. P. (2009). Methods for equivalence and noninferiority testing. Biol. Blood Marrow Transplant. 15, 120–127. 10.1016/j.bbmt.2008.10.004 19147090 PMC2701110

[B9] Davison A. C. Hinkley D. V. (1997). Bootstrap Methods and Their Applications. Cambridge: Cambridge University Press.

[B10] de Los Campos G. Gianola D. Rosa G. J. Weigel K. A. Crossa J. (2010). Semi-parametric genomic-enabled prediction of genetic values using reproducing kernel hilbert spaces methods. Genet. Res. 92, 295–308. 10.1017/S0016672310000285 20943010

[B11] Falconer D. S. Mackay T. F. C. (1996). Introduction to Quantitative Genetics. Essex: Longman Group.

[B12] Friedman J. Hastie T. Tibshirani R. (2001). The Elements of Statistical Learning, Vol. 1. New York, NY: Springer Series in Statistics New York.

[B13] Gelman A. Shalizi C. R. (2013). Philosophy and the practice of bayesian statistics. Br. J. Math. Stat. Psychol. 66, 8–38. 10.1111/j.2044-8317.2011.02037.x 22364575 PMC4476974

[B14] Gianola D. de Los Campos G. Hill W. G. Manfredi E. Fernando R. (2009). Additive genetic variability and the bayesian alphabet. Genetics 183, 347–363. 10.1534/genetics.109.103952 19620397 PMC2746159

[B15] Glaubitz J. C. Casstevens T. M. Lu F. Harriman J. Elshire R. J. Sun Q. . (2014). Tassel-gbs: a high capacity genotyping by sequencing analysis pipeline. PLoS ONE 9:e90346. 10.1371/journal.pone.0090346 24587335 PMC3938676

[B16] Habier D. Fernando R. L. Kizilkaya K. Garrick D. J. (2011). Extension of the bayesian alphabet for genomic selection. BMC Bioinformatics 12, 1–12. 10.1186/1471-2105-12-186 21605355 PMC3144464

[B17] Henderson C. R. (1984). Applications of Linear Models in Animal Breeding. Guelph, ON: University of Guelph.

[B18] Heslot N. Yang H.-P. Sorrells M. E. Jannink J.-L. (2012). Genomic selection in plant breeding: a comparison of models. Crop. Sci. 52, 146–160. 10.2135/cropsci2011.06.0297

[B19] Hothorn T. Leisch F. Zeileis A. Hornik K. (2005). The design and analysis of benchmark experiments. J. Comput. Graph. Stat. 14, 675–699. 10.1198/106186005X59630

[B20] Martini J. W. Gao N. Cardoso D. F. Wimmer V. Erbe M. Cantet R. J. . (2017). Genomic prediction with epistasis models: on the marker-coding-dependent performance of the extended gblup and properties of the categorical epistasis model (ce). BMC Bioinformatics 18, 1–16. 10.1186/s12859-016-1439-1 28049412 PMC5209948

[B21] Martini J. W. Wimmer V. Erbe M. Simianer H. (2016). Epistasis and covariance: how gene interaction translates into genomic relationship. Theor. Appl. Genet. 129, 963–976. 10.1007/s00122-016-2675-5 26883048

[B22] Meuwissen T. H. Hayes B. J. Goddard M. E. (2001). Prediction of total genetic value using genome-wide dense marker maps. Genetics 157, 1819–1829. 10.1093/genetics/157.4.1819 11290733 PMC1461589

[B23] Ober U. Huang W. Magwire M. Schlather M. Simianer H. Mackay T. F. (2015). Accounting for genetic architecture improves sequence based genomic prediction for a drosophila fitness trait. PLoS ONE 10:e0126880. 10.1371/journal.pone.0126880 25950439 PMC4423967

[B24] Perez P. de los Campos G. (2014). Genome-wide regression and prediction with the bglr statistical package. Genetics 198, 483–495. 10.1534/genetics.114.164442 25009151 PMC4196607

[B25] R Core Team (2021). R: A Language and Environment for Statistical Computing. Vienna: R Foundation for Statistical Computing.

[B26] Runcie D. Cheng H. (2019). Pitfalls and remedies for cross validation with multi-trait genomic prediction methods. G3 9, 3727–3741. 10.1534/g3.119.400598 31511297 PMC6829121

[B27] Schrauf M. F. Martini J. W. Simianer H. de Los Campos G. Cantet R. Freudenthal J. . (2020). Phantom epistasis in genomic selection: on the predictive ability of epistatic models. G3 10, 3137–3145. 10.1534/g3.120.401300 32709618 PMC7466977

[B28] Sorensen D. Gianola D. (2002). Likelihood, Bayesian and MCMC Methods in Quantitative Genetics. New York, NY: Springer.

[B29] Thompson R. (2019). Desert island papers—a life in variance parameter and quantitative genetic parameter estimation reviewed using 16 papers. J. Anim. Breed. Genet. 136, 230–242. 10.1111/jbg.12400 31247681

[B30] VanRaden P. M. (2008). Efficient methods to compute genomic predictions. J. Dairy Sci. 91, 4414–4423. 10.3168/jds.2007-0980 18946147

[B31] Wang W. Mauleon R. Hu Z. Chebotarov D. Tai S. Wu Z. . (2018). Genomic variation in 3,010 diverse accessions of asian cultivated rice. Nature 557, 43–49. 10.1038/s41586-018-0063-9 29695866 PMC6784863

[B32] Whittaker J. C. Thompson R. Denham M. C. (2000). Marker-assisted selection using ridge regression. Genet. Res. 75, 249–252. 10.1017/S0016672399004462 10816982

